# The Effectiveness of Intraocular Methotrexate in the Treatment of Posterior Uveitis in Behçet's Disease Patients Compared to Retrobulbar Steroids Injection

**DOI:** 10.1155/2016/1678495

**Published:** 2016-12-13

**Authors:** Hossam El Din Mohamed Khalil, Heba A. El Gendy, Hala Ahmed Raafat Youssef, Hazem Effat Haroun, Tamer Atef Gheita, Hossam Mahmoud Bakir

**Affiliations:** ^1^Beni Suef University, Beni Suef, Egypt; ^2^Cairo University, Giza, Egypt

## Abstract

*Aim of Work*. To evaluate the efficacy of intravitreal methotrexate (MTX) compared to retrobulbar triamcinolone acetonide (TAA), in controlling posterior segment involvement and inducing remissions among Behçet's disease (BD) patients.* Study Design*. This is a cross-sectional nonrandomized comparative study.* Patients and Methods*. 31 adult BD male patients with a mean disease duration of 5.45 years who presented with bilateral posterior segment involvement were included. Each patient received intravitreal injection of 400 *μ*g/0.1 mL (MTX) for the right eye (Group A) and 1 mL of retrobulbar 40 mg/mL TAA for the left eye (Group B).* Results*. 90% of eyes showed complete improvement of anterior chamber reaction, whereas an improvement in vitreous activity in 77% with no significant differences between both groups (*p* ≤ 0.1). BCVA improved in 77.4% eyes (Group A) compared to 87.1% (Group B) (*p* ≤ 0.4). Relapses were noted in 11 eyes (35.5%), in group A, with the mean duration of remission being 19.1 weeks ± 2.13 compared to 7.35 ± 2.8 in 20 eyes (64.5%) in group B (*p* ≤ 0.1).* Conclusion*. No statistical differences were found between both treatment modalities; however, based on clinical observations, intravitreal MTX may ensure better control of inflammatory reaction and may encourage longer remission as compared to retrobulbar TAA in BD patients.

## 1. Introduction

Behçet's disease (BD) is a chronic inflammatory, multisystem disorder that was first reported in 1930s by the Turkish dermatologist H Behçet [1]. The exact pathogenesis is not well established; however, an autoimmune response in genetic predisposed individuals has been postulated. Moreover, a triggering infectious agent might be attributed to the alteration of the immune response in affected patients, whereas vasculitis has been considered the cornerstone of the condition, with superimposed coagulation and thrombotic disorders [2]. The condition, however, is characterized by recurrent oral and genital ulcers, ocular inflammation, CNS involvement, and positive pathergy test results [3, 4].

Ocular involvement in the form of chronic relapsing-remitting anterior and/or posterior uveitis has been reported in 60%–80% of patients with Behçet's disease, with more male predilection in the Middle East, with a reported ratio of 2 : 1, and the tendency of the disease to be more severe in the affected males as compared to the female patients [5, 6].

Different treatment modalities have been proposed for controlling the ocular involvement and vision threatening complications in BD patients, whereas corticosteroids remain the cornerstone for treatment in the majority of cases. Moreover, the use of immunosuppressive drugs has been advocated in selected cases, which might result in controlling the disease process and inducing long term remissions [7].

The systemic use of these drugs carries the risk of systemic side effects and hence the postulation of intravitreal approach as well as the periocular route of administration in cases with ocular involvement, aiming at direct delivery of the drug to the target organ at a satisfactory concentration as well as avoiding systemic side effects of the drug [8, 9].

Methotrexate (MTX) is an antimetabolite that was proposed at low doses for the treatment of rheumatoid arthritis (RA) and ocular inflammatory conditions with satisfactory results [10].

Intravitreal MTX was first introduced for the treatment of recurrent intraocular lymphoma, which was noticed to induce prolonged local remission of ocular disease even with an aggressively growing tumor, with subsequent use in cases with chronic inflammatory ocular conditions [8].

In the current study, the efficacy of intravitreal methotrexate in controlling posterior segment involvement and inducing remission in BD patients, compared to retro bulbar steroids injection, was evaluated.

## 2. Patients and Methods

The study was done in accordance with the ethical standards of the Declaration of Helsinki 1964 [11] and institutional ethical committee policy. All patients were requested to sign a formed consent before participation in the current study with a full declaration of the intervention and possible complications.

In this cross-sectional nonrandomized comparative study, 31 adult BD male patients aged 22–45 years (29.7  years  ± 6.17  SD) who presented with bilateral almost symmetrical active posterior uveitis, with a disease duration of 2–12 (mean 5.45 ± 2.39) years were enrolled from patients attending the Rheumatology and Rehabilitation outpatient clinic, in-patient department, as well as those attending the Ophthalmology outpatient clinics, Faculty of Medicine, Cairo University Hospitals, from the period of February 2014 to December 2015. The diagnosis of BD was established to meet the set of diagnostic criteria published by the International Study Group for Behçet's disease in 1990 [3].

A full history taking including the disease manifestations, duration of the disease, and previous medications received, as well as current medications, was taken for all participants, whereas current medications received by the patients were considered and patients receiving corticosteroids and/or immunosuppressant therapy for management of their disease were not excluded. Moreover, all enrolled patients were further retrospectively traced regarding their treatment regimens throughout the 3-month duration prior to their recruitment making sure that no recent alteration in the treatment protocol was applied, in order to exclude the impact of systemic drug modulation on the ocular results throughout the study period.

Full general examination and skin pathergy test were carried out for all patients, to establish the diagnosis of the disease.

Blood specimens were collected after an overnight fast, for complete blood count, liver function tests, platelets count, erythrocyte sedimentation rate, antinuclear antibody level, and renal function tests, prior to the initiation of intravitreal and/or retrobulbar therapy, as well as chest X-ray to exclude chest infection or TB.

### 2.1. Ophthalmological Examination

All patients underwent complete ophthalmological examination, that is, best corrected visual acuity (BCVA) testing, anterior segment examination, slit lamp biomicroscopy, tonometry, and indirect ophthalmoscopy, whereas other investigations including fundus photography, fluorescein angiography, and OCT were performed as indicated.

Patients with other ocular pathologies, end-stage disease with no light perception attributable to retinal ischemia and/or optic atrophy, bilateral irreversible blindness, ocular hypertension, or glaucoma, as well as concurrent active infection, were excluded from the current study.

### 2.2. Exposure

The patients were nonrandomly scheduled for intravitreal methotrexate (MTX) injection in their right eyes (*Group A*) and retrobulbar triamcinolone actenoide (TAA) injection for the left eyes (*Group B*). Injection was performed under full aseptic conditions, with topical benoxinate HCl 0.4% drops instilled before injection.

In* Group A* eyes, 400 *μ*g/0.1 mL of MTX was injected intravitreally via the pars plana, 3.5 to 4.0 mm posterior to the inferotemporal limbus using a 27-gauge needle, whereas 1 mL of 40 mg/mL Sterile TAA Suspension (Kenakort-A IM, Bristol-Meyers Squibb Company, Egypt) mixed with 0.5 mL of 2% lignocaine (lidocaine) was slowly injected retrobulbarly, using a 1.25-inch 23-gauge needle, for* Group B* eyes.

For retrobulbar injection, the needle was inserted at the inferotemporal aspect of the lower lid directed towards the orbital apex, and the patient was asked to look straight ahead while the globe was pushed towards the superior orbit with the index finger of the noninjecting hand; thus, a quick pass of the needle, in the absence of any eye movement, assures nonpenetration of the globe.

### 2.3. Follow-Up

Patients were examined one week after injection and monthly thereafter for a period of 6 months. At each visit, measurement of the best corrected visual acuity (BCVA) and intraocular pressure (IOP) was made. Slit lamp biomicroscopy and dilated fundoscopy to view individual cells within the vitreous cavity and assessment of posterior segment were done.

### 2.4. Remission and Relapses

Remissions were considered with <1+ cells in the anterior chamber or vitreous, whereas relapses were defined as the development of ≥1+ cells in the anterior chamber or vitreous cavity.

### 2.5. Statistical Analysis

Data were coded and analyzed using the statistical package SPSS version 15 for Windows. Data were summarized using mean ± standard deviation (SD) for quantitative variables and frequency and percentage for qualitative variables. Significant differences were calculated using Student's* t*-test and Mann–Whitney* U* test for continuous variables. Chi square (*χ* ^2^) test with Yates' correction or Fisher's exact tests was used for comparing categorical data, whereas *p* value < 0.05 was considered significant.

## 3. Results

In this cross-sectional nonrandomized comparative study, 31 adult BD male patients with a mean age of 29.7 years ± 6.17 and a mean disease duration of 5.45 years ± 2.39 who presented with nearly symmetrical bilateral posterior segment involvement were enrolled.

### 3.1. Preinjection Data

Anterior chamber reaction in the form of flare and cells ranging from + to ++ was documented in 20 (64.5%) eyes in* Group A* and 18 (58%) eyes in* Group B*, whereas vitreous cells were detected in 25 (80.6%) and 22 (70.9%) eyes, respectively (*p* ≤ 0.4).

For* Group A*, the intraocular pressure values (IOP) ranged within 12–18 mmHg with a mean of 13.9 ± 1.68 (SD), as compared to values ranging within 10–18 mmHg (mean 13.93 ± 2.2) in* Group B*, which was noted as being statistically insignificant (*p* ≤ 0.39).

The preinjection BCVA ranged within 0.1–0.5 with a mean of 0.26 ± 0.12 for* Group A*, as compared to 0.125–0.6 (mean 0.3 ± 0.12) for* Group B*, which was also considered of no* t* statistical significance (*p* ≤ 0.4).

### 3.2. Postinjection Data

In eyes with preinjection anterior chamber activity (38 eyes), an improvement of anterior chamber activity was achieved in all eyes after injection throughout the scheduled follow-up visits, whereas 90% of eyes showed complete improvement within the first 2 weeks after injection, whereas no significant differences were noted regarding either group (*p* ≤ 0.1).

An improvement in vitreous activity was achieved in 47 eyes, with complete resolution in 10 eyes by the end of 1st week, 12 eyes by the end of the 2nd week, 9 eyes by the end of 1st month, and 12 eyes at 2 months after injection, whereas 4 eyes (8.5%) failed to achieve complete resolution of vitreous reaction by the end of the follow-up period, again with no differences between either group of eyes (*p* ≤ 0.1).

No postinjection elevation of IOP was detected in any of the eyes injected with intravitreal MTX (*Group A*), although a transient elevation of IOP was recorded in 2 (6%) of the retrobulbar TAA injected eyes (*Group B*) in the early follow-up period, that is, 1st week, which was controlled with topical combination of *β* blockers and prostaglandin analogues drops for 4–6 weeks' duration.

Therefore, in* Group A*, the mean preinjection IOP was 13.9 mmHg ± 1.68 (SD), as compared to a mean postinjection value of 14.06 mmHg ± 1.62 (SD), with no statistical differences between both values (*p* ≤ 0.1).

Meanwhile, in* Group B*, despite that transient elevation of IOP, the mean pre- and postinjection values were 13.93 ± 2.2 and 14.74 ± 3.28, respectively, which were found to be of no statistical significant, within the same group as well as compared to the postinjection values in the other group (*p* ≤ 0.03).

Moreover, despite that IOP elevation, no other complications were encountered in either group of eyes during the injection procedure or throughout the follow-up period.

A nonsignificant improvement of BCVA was recorded in 24 eyes (77.4%) in MTX injected eyes, that is, 0.23 ± 0.12  &  0.42 ± 0.09, respectively (*p* ≤ 0.4), compared to a mean improvement of 0.29 ± 0.1  &  0.42 ± 0.08 in 27 eyes (87.1%) injected with retrobulbar TAA, which was considered of no significant difference neither for the tested group nor for the comparison between eyes in both groups (*p* ≤ 0.2  &  0.4), respectively, as shown in Table 1, whereas the maximum improvement and stabilization were noticed to be achieved around the 3rd month after injection.

A considerable improvement in ocular inflammatory reaction as well as visual acuities has been achieved in 58 eyes (93.5%) in both groups throughout the follow-up period, with no significant differences between both groups (*p* ≤ 0.3); however, relapses that required reinjection were noted in 11 eyes (35.5%), in* Group A* during the follow- up at a duration ranging from 22 to 16 weeks postinjection (mean 19.1 ± 2.13), compared to 20 eyes (64.5%) in Group B at a period ranging from 14 to 4 weeks with a mean value of 7.35 ± 2.8, with no significant differences between both groups (*p* ≤ 0.1).

## 4. Discussion

Behçet's disease is a chronic noninfectious condition with ocular involvement in up to 80% of cases, which are characterized by being chronic with a remitting course, whereas the main goal of treatment is to induce and maintain a long duration remission of the disease condition [12].

Periocular and intravitreal drug delivery have been proposed in a trial to overcome the potential side effects of systemic drugs used as well as to insure a high concentration level of the drug delivered to the target organ for superior therapeutic effect [13].

In the present study, the effect of intravitreal MTX injection was compared with retrobulbar corticosteroid injection in cases with posterior segment involvement with BD, regarding the control of inflammatory manifestations as well as the induction of remission.

Reviewing literature, the efficacy of retrobulbar corticosteroids in ocular inflammatory conditions was noted to be evaluated by many investigators, with satisfactory results in chronic cases. Meanwhile, limited publications regarding the role of intravitreal MTX in intraocular inflammatory conditions and no published comparative studies regarding both modalities were retrieved [8, 14–17].

In the present study, 31 adult male patients with established BD and bilateral posterior segment involvement were intravitreally injected with 400 *μ*g/0.1 mL of MTX, for their right eyes (*Group A*), and were compared to (*Group B*) with 1 mL of 40 mg/mL retrobulbar Sterile TAA Suspension slowly injected for the other left eyes.

In the present study, an improvement of anterior and posterior segment activity was achieved in all cases, with complete resolution of anterior segment reaction in 90% of cases during the first 2 weeks, whereas posterior segment resolution was achieved in 93.6% of eyes by the end of the 2nd month after injection.

Our results are comparable to those reported by Okada et al. [18], with a reported clinical efficacy of 96% for vitritis, 82% for CME, and 33% for posterior retinal vasculitis after trans-tenon retrobulbar TA infusion, and Gamal et al. [19], who recorded 93% and 86% rate of complete resolution (24 out of 26 eyes) regarding ant chamber and vitreous activities, respectively, following ultrasonographic verified retrobulbar TAA injection.

Moreover, the observed improvement of inflammatory activity may be explained based on previous published data with documented significant improvement of anterior chamber flare and reduced protein accumulation by laser flare photometric studies following orbital floor triamcinolone acetonide injections, as well as the decreased intraocular cytokine levels after intravitreal MTX in cases with refractory inflammatory reactions [20, 21].

77.4% of eyes with intravitreal MTX achieved an improvement of the mean BCVA from 0.23 ± 0.12 to 0.42 ± 0.09, with the maximum improvement and stabilization noted at a median duration of 3 months after injection, comparable with the reported data by Taylor et al. [22], who documented a significant improvement VA of 4–4.5 lines following intravitreal MTX injection in 15 eyes with BD at a follow-up period of 3–6 months, respectively.

Again our data are still comparable with those recorded by Bae and Lee [20], as they reported a mean improvement of VA by 3 or more lines from baseline measures at a follow-up period of 24 ± 8.2 weeks after monthly intravitreal MTX injection with a mean of 4.3 ± 1.0 injections in 85.7% of the injected eyes in their case series of seven eyes of seven patients with Bechet disease.

Furthermore, we noted a mean improvement of BCVA from 0.29 ± 0.1 to 0.42 ± 0.08 in 27 eyes (87.1%) injected with retrobulbar TAA throughout the follow-up period, again with the maximum improvement and stabilization achieved around the 3rd month after injection, which is comparable with the mean improvement documented by other investigators [19, 23, 24].

In our study, no significant changes in the mean IOP values were noted after injection in* Group A* with intravitreal MTX, with pre- and postinjection values of 13.9 mmHg ± 1.68 and 14.06 mmHg ± 1.62, respectively; however, due to limited publications regarding the IOP changes with intravitreal MTX, the comparison of data was abounded, as only one eye with an elevated IOP > 21 mmHg was reported in a case series of 38 eyes after intravitreal MTX injection [15].

Moreover, 2 eyes showed a transient elevation of IOP following retrobulbar TAA injection, at a rate of 6%, which was controlled within 4–6 weeks with no significant alterations in IOP values postinjection by the end of our scheduled follow-up.

In the present work, the incidence of ocular hypertension was considered the least as compared with other published data following retrobulbar steroid injection, which may be attributed to meticulous patient selection in the present study, as cases with ocular hypertension or previous glaucoma were excluded [18, 21, 25].

Relapses that required reinjection were noted in 11 eyes after MTX injection after a mean duration of 19.1 ± 2.13 at a rate of 35.5%, which were compared to previous published data, with reported relapses at 33.3% after a median of 4 months after injection, and a rate of 27% after 3-4 months' duration [15, 16].

Meanwhile, in* Group B* eyes, relapses were recorded in 20 eyes at a rate of 64.5% after a mean duration of 7.35 ± 2.8 weeks, which is comparable with published data, with the duration of action of TAA expected to last for 4–6 weeks after being injected in the posterior subtenon and the possibilities of second attacks thereafter with the recommendation of reinjection after 4 weeks after injection [26].

Furthermore, our results are not comparable with published results by Gamal et al. [19], who reported reattacks in 19% of their cases at a duration ranging from 3 to 6 months, with lower rates of relapses and longer duration of remission, which may be attributed to the use of ultrasonography technique to verify their retrobulbar TAA injection, which may ensure a precise localization of the drug with subsequent better results.

In the present study, no significant statistical differences were elicited between either treatment modalities; however, based on our clinical observations, the lower incidence of relapses as well as the longer duration of remission noted after intravitreal MTX injection may encourage this treatment modality over retrobulbar TAA injection.

Furthermore, the risk of IOP elevation after retrobulbar injection may still favor intravitreal MTX injection in ocular BD.

## 5. Conclusion

Despite being less invasive than the intravitreal approach, the efficacy of retrobulbar TAA injection may be questioned, being a blind procedure that may not ensure a direct concentration of the drug at the target area, for example, the macular region in cases with CME, as well as the increasing incidence of IOP elevations; however, intravitreal injection may ensure adequate delivery of the drug to the eye.

Intravitreal MTX, despite being more invasive, however, may ensure better control of inflammatory reaction and may encourage longer remission as compared to retrobulbar TAA, with decreasing risk of IOP elevation. Further studies on a larger number of patients and longer follow-ups are recommended to ensure the efficacy of intravitreal MTX in controlling posterior uveitis in BS patients.

## Figures and Tables

**Table 1:** The pre- & post-injection improvement of BCVA.

	*Group A*		*Group B*		*p* value
	Pre-injection	Post-injection	Pre-injection	Post-injection	Between groups
Min	0.1	0.2	0.125	0.3	
Max	0.5	0.6	0.5	0.6	≤*0.4*
Mean	0.23 ± 0.12	0.42 ± 0.09	0.29 ± 0.1	0.49 ± 0.08	
*p* value	≤*0.4*		≤*0.2*		
